# Accumulation of radiocarbon in ancient landscapes: A small but significant input of unknown origin

**DOI:** 10.1038/s41598-023-34080-4

**Published:** 2023-05-08

**Authors:** Adrian Broz, Jerod Aguilar, Xiaomei Xu, Lucas C. R. Silva

**Affiliations:** 1 Department of Earth, Atmospheric and Planetary Sciences, Purdue University, West Lafayette, IN 47907 USA; 2 Department of Earth Sciences, University of Oregon, Eugene, OR 97403 USA; 3 Department of Earth Sciences, Southern Methodist University, Dallas, TX 97403 USA; 4 University of California Irvine, Irvine, CA 92697 USA; 5 Department of Geography, University of Oregon, Eugene, OR 97403 USA; 6 Environmental Studies, Department of Biology, Institute of Ecology and Evolution, University of Oregon, Eugene, OR 97403 USA

**Keywords:** Biogeochemistry, Environmental sciences, Solid Earth sciences

## Abstract

The persistence of organic carbon (C) in soil is most often considered at timescales ranging from tens to thousands of years, but the study of organic C in paleosols (i.e., ancient, buried soils) suggests that paleosols may have the capacity to preserve organic compounds for tens of millions of years. However, a quantitative assessment of C sources and sinks from these ancient terrestrial landscapes is complicated by additions of geologically modern (~ 10 Ka) C, primarily due to the infiltration of dissolved organic carbon. In this study, we quantified total organic C and radiocarbon activity in samples collected from 28- to 33-million-year-old paleosols that are naturally exposed as unvegetated badlands near eastern Oregon’s “Painted Hills”. We also used thermal and evolved gas analysis to examine the thermodynamic stability of different pools of C in bulk samples. The study site is part of a ~ 400-m-thick sequence of Eocene–Oligocene (45–28 Ma) paleosols, and thus we expected to find radiocarbon-free samples preserved in deep layers of the lithified, brick-like exposed outcrops. Total organic C, measured in three individual profiles spanning depth transects from the outcrop surface to a 1-m depth, ranged from 0.01 to 0.2 wt% with no clear C-concentration or age-depth profile. Ten radiocarbon dates from the same profiles reveal radiocarbon ages of ~ 11,000–30,000 years BP that unexpectedly indicate additions of potentially modern organic C. A two-endmember mixing model for radiocarbon activity suggests that modern C may compose ~ 0.5–2.4% of the total organic C pool. Thermal and evolved gas analysis showed the presence of two distinct pools of organic C, but there was no direct evidence that C compounds were associated with clay minerals. These results challenge the assumption that ancient badland landscapes are inert and “frozen in time” and instead suggest they readily interact with the modern C cycle.

## Introduction

Decades of work have shown that ancient, buried soils (paleosols) can preserve soil organic carbon (C) over geological timescales. Organic carbon compounds in Archean (~ 2 Ga) paleosols include carbonaceous microfossils and filamentous organic structures^1,2^ that likely originated from cyanobacterial mats that lived on the soil surface^3^. Organic-walled fungal microfossils have also been found in Proterozoic (~ 1 Ga) terrestrial environments^4^. Geologically younger paleosols contain pyrogenic carbon^5,6^, carbonaceous root traces^7,8^, and carbonaceous compression fossils of plants that grew in soils millions of years ago^9^. The examination of organic matter preservation in paleosols suggests that terrestrial (nonmarine) environments may preserve organic matter for millions or even billions of years, though at concentrations that are orders of magnitude lower than modern soils^10^, likely due to diagenetic losses after burial^10^.

Outstanding questions remain about types and sources of organic molecules in paleosols. How much of the carbon is original to the soil? Are there contributions from organic carbon sourced from the modern biosphere, and can interactions with the modern C cycle release ancient C from exhumed paleosols^11^? Furthermore, few estimates exist for the rates of modern C accumulation in ancient terrestrial landscapes such as badlands, which are often sequences of naturally exhumed paleosols^12^. When buried soils are naturally exposed to the modern weathering zone, such as in modern badlands, ancient C compounds can be oxidized by modern biogeochemical processes and ultimately returned to the atmosphere^13^. The pool of ancient carbon in exhumed terrestrial landscapes is not readily considered in terrestrial carbon budgets because it is difficult to measure^13^, and therefore quantifying the capacity of ancient soils to preserve and/or cycle ancient organic C back to the atmosphere is not well constrained.

One challenge to a comprehensive understanding of C sources and sinks in ancient soils is widespread and pervasive addition of C compounds from the modern biosphere. Here we refer to “modern” C as post-bomb organic C compounds (> 1950) and distinguish it from the geological definition of modern (~ 10,000 years). Additions of modern C to ancient samples may inflate estimates of so-called “preserved” C compounds in paleosols. This contamination by modern organic carbon can ultimately confound efforts to understand organic matter persistence in ancient soils, in part because it is difficult to quantify the sources and types of organic carbon in soils that are millions of years old. In other words, additions of modern carbon to paleosols can inflate estimates of the so-called “preserved” carbon. For example, carbon from microbial biomass and/or plant root exudates from the modern weathering zone can leach downwards and accumulate in paleosols (e.g.,^14^). Eolian and groundwater-driven deposition of allochthonous C are additional possibilities^15^, though groundwater found in bedrock can be depleted in modern C^16^. Therefore, a method to constrain the sources and approximate ages of organic carbon in bulk paleosol samples would provide a valuable technique to understand if and how modern carbon tends to accumulate in ancient exhumed landscapes.

Radiocarbon dating of the organic fraction in soils^17^ and paleosols^14^ can provide valuable constraints on the age of C compounds present, but is not typical for radiocarbon dating to be applied to Cenozoic (~ 30 Ma) paleosols because it is assumed that they are free of radiocarbon. As a result, most studies of paleosol radiocarbon are focused on soils of Quaternary age or younger^14,18–20^. It therefore remains undetermined if much older paleosols commonly contain radiocarbon. A widespread and pervasive modern biosphere would presumably increase radiocarbon activity with surface layers of ancient exhumed soils. However, digging deeper into lithified paleosol outcrops that are exposed at the surface (e.g., excavating to a 1-m depth) may increase the likelihood that radiocarbon-free C can be found, which was the motivation for this study. Discovery of radiocarbon free material in deep samples would imply that ancient lithified soils, now brick-like claystones, are somewhat insulated from the modern weathering zone and thus possibly insulated from interactions with the modern C cycle.

**Figure 1:**
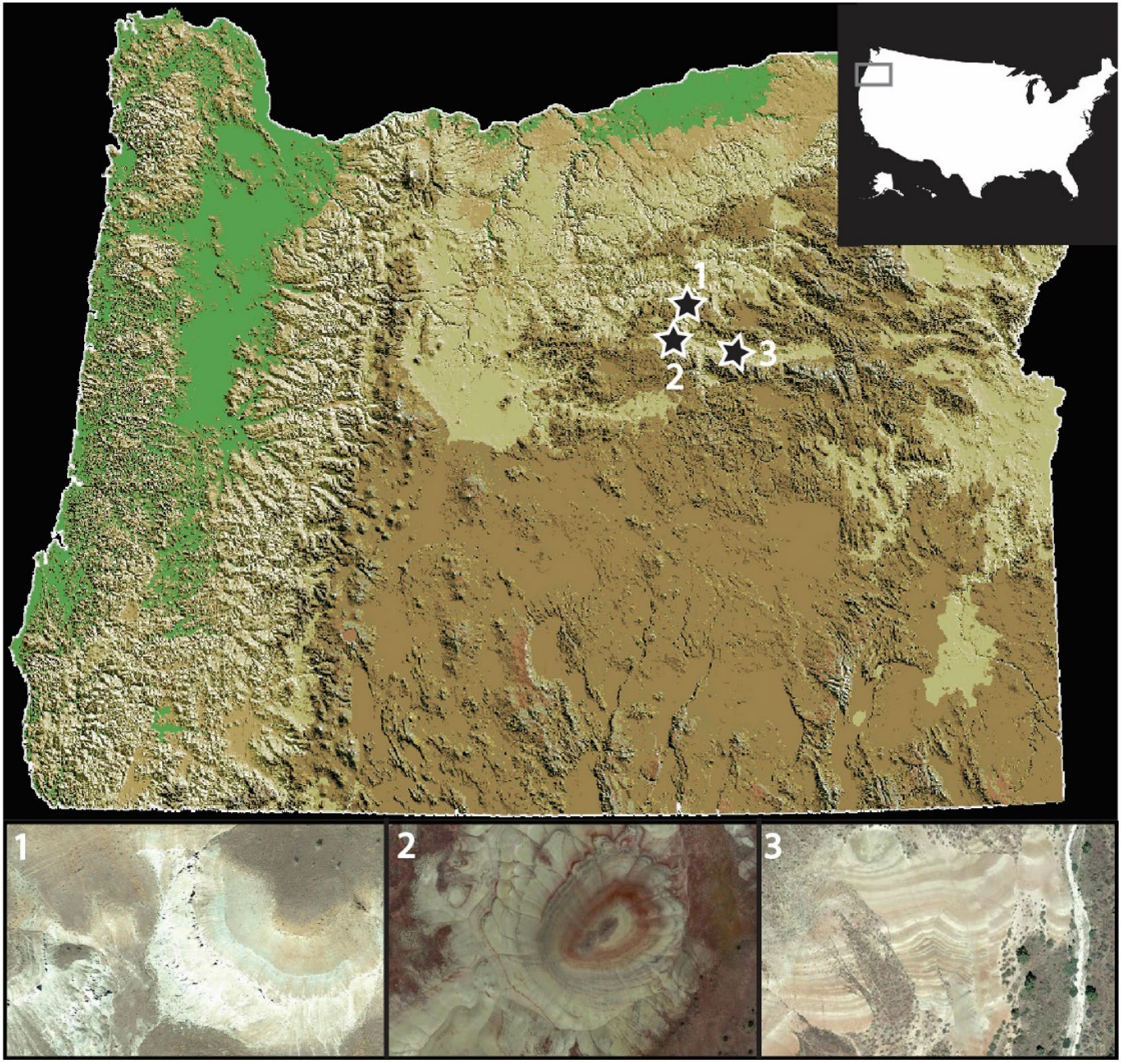
Sampling sites from three ancient terrestrial landscapes near the John Day Fossil Beds National Monument, eastern Oregon, USA. **1**, 28-million-year-old paleosols from the lower Turtle Cove Member of the John Day Formation; **2** and **3**, 33-million-year-old paleosols from the middle Big Basin member of the John Day Formation, near the local Eocene–Oligocene boundary (~ 33 Ma). Map and satellite images were created using ArcGIS Pro 3.1 (https://www.esri.com).

In this work, we tested the hypothesis that unvegetated and lithified 28- to 33-million-year-old paleosols contain small amounts of ancient radiocarbon-dead materials. We evaluated the total organic carbon content of three paleosol profiles from eastern Oregon (Fig. 1) and performed radiocarbon analysis on bulk samples that were collected from the outcrop surface to a horizontal depth of 1-m into each outcrop. To quantify potential additions of modern organic carbon as a function of horizontal depth into the outcrop, we employed a two-endmember isotopic mixing model to estimate the percentage of modern carbon in ancient samples, a technique commonly applied in studies of terrestrial C dynamics^21,22^. A mixing model approach can help constrain the amount and isotopic composition of C compounds that may have been added to paleosols. Lastly, we used thermal and evolved gas analysis to evaluate the thermodynamic stability of organic carbon in several samples. Thermal analysis techniques such as EGA have been employed for understanding the nature and stability of organic matter in modern soils^23–25^, though at present there are limited studies of paleosols. While pyrolysis EGA does not provide insight into the specific types of organic compounds present, it can provide information about the thermodynamic stability of organic carbon^23,26^, and whether organic compounds are associated with minerals such as phyllosilicates and sulfates^27^. We assessed evolutions of H_2_O, CO_2_ and organic fragments in bulk paleosol samples to constrain whether organic compounds were primarily associated with clay minerals or other hydrated phases. The objectives of this work were to (A) determine whether 28 to 33-million-year-old paleosols contain radiocarbon at depth; (B) constrain the amount of modern carbon that could have accumulated in ancient samples; and (C) determine if organic carbon, whether ancient or modern, was associated with clay minerals.

Many of the Oregon paleosol profiles formed as a result of sustained pedogenic alteration of rhyodacitic to andesitic volcanic ash and tuff that was periodically emplaced by nearby ancient stratovolcanoes from ~ 45 to 26 Ma^12^. A detailed examination of morphology, mineralogy and chemistry of approximately 40 individual paleosol profiles spanning nearly 500 m of stratigraphy is reported in^9^. The eastern Oregon field site has also been previously considered as a “Mars-analog” because the mineralogy and geochemistry of the paleosol profiles resemble highly altered sedimentary rocks on Mars that are approximately 3.7–4.1 billion years old^28–30^. Past work has examined the mineralogy, diagenesis, and organic preservation potential of several of these profiles (Fig. 1) for comparisons with Mars^30,31^. Previously, radiocarbon dating of four samples collected from shallow depths (< 40 cm) into a ~ 33 million year old paleosol outcrop revealed conventional radiocarbon ages of ~ 7000–14,500 years BP, suggesting there had been inputs of radiocarbon into the lithified, brick-like samples^31^ (Table S1). This unanticipated finding motivated the present work for radiocarbon dating of deeper samples across three additional profiles separated by space and time.

## Methods

### Sample collection

**Figure 2:**
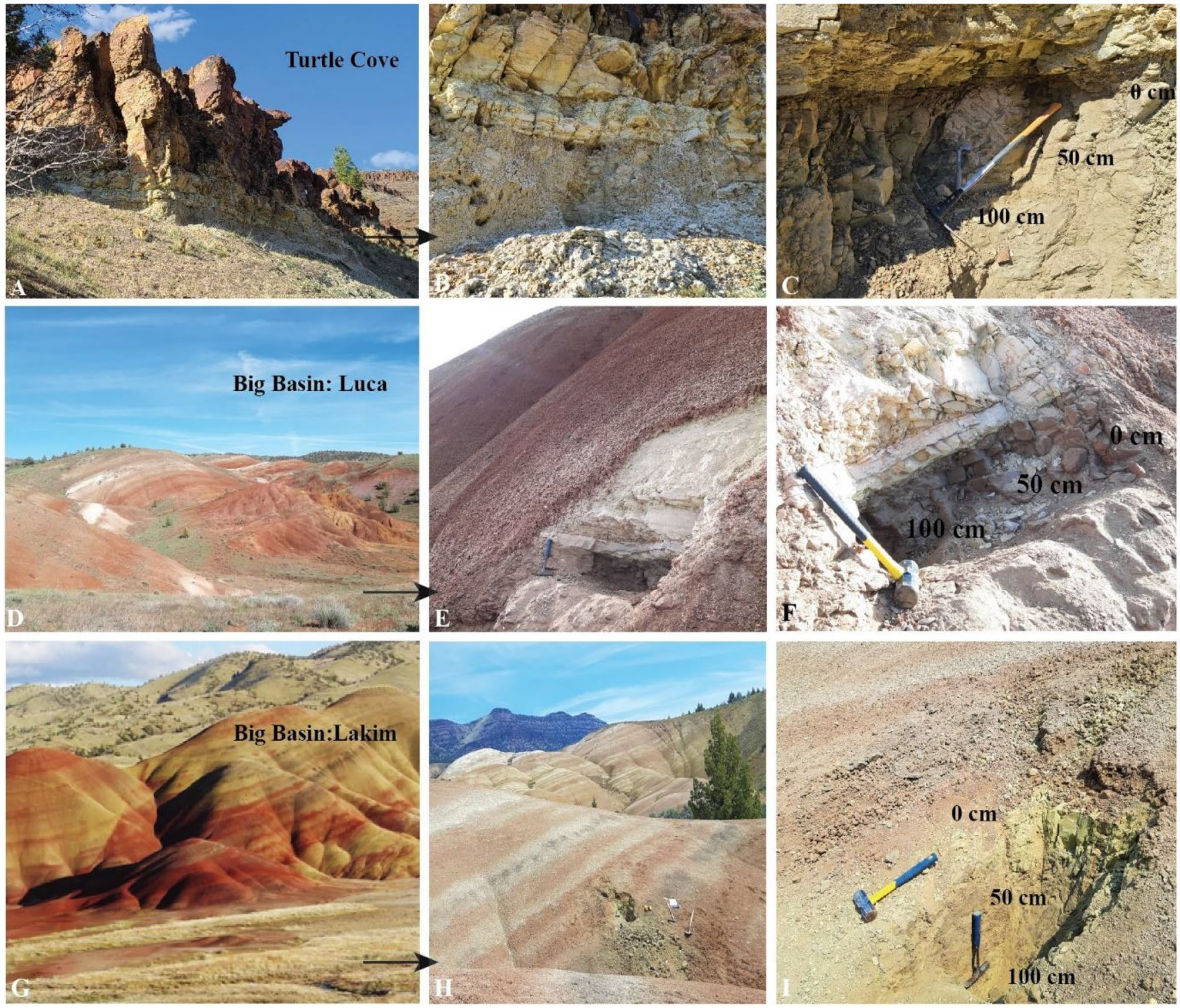
Paleosol outcrops examined in this work. (**A**), Oligocene (28 Ma) lower Turtle Cove Member of the John Day Formation (44.670229, − 119.627418); (**B**) and (**C**), Allophane/imogolite-rich Andisol paleosols buried by overlying ignimbrite (horizontal tan and brown layers); (**D**) Early Oligocene (33 Ma) middle Big Basin Member of the John Day Formation (44.628605, − 120.212263); (**E**) and (**F**), Al and Fe-smectite-rich Alfisol paleosol (“Luca” pedotype from^9^) buried by volcanic tuff (sharp white layer); **G)** Early Oligocene (33 Ma) middle Big Basin Member of the John Day Formation (44.638333, − 120.220021); (**H**) and (**I**), Al/Fe smectite and Mn-oxide-bearing Inceptisol paleosol (“Lakim” pedotype from^9^) buried by gleyed biotite-bearing tuff (green layer).

Large (0.5 kg) lithified hand samples were collected from three paleosol outcrops within the Eocene–Oligocene (33–26 Ma) John Day Formation in eastern Oregon (Fig. 2). Two of the outcrops (“Luca” and “Lakim”), are from the late Eocene (~ 33 Ma) middle Big Basin Member of the John Day Formation, and one (“Turtle Cove”), is from the Oligocene (28 Ma) lower Turtle Cove Member of the John Day Formation^12^.

Several measures were taken during sample collection to minimize organic contamination. All sampling materials (glassware, foil, mortar and pestle) were ashed in a muffle furnace for 24 h at 550 °C and wrapped in aluminum foil prior to arriving at the field site. During collection, all samples were collected in glass vials and capped with aluminum foil prior to securing the plastic vial lids. An ashed sample of silica sand served as a method-level blank and was carried through as many of the sampling steps as possible. Nitrile gloves were worn during all stages of sample collection. Rock hammers and chisels were flamed with isopropyl alcohol for 3–5 min prior to sample collection in order to ensure these materials were sterile.

After removing the surface soil and exposing the lithified paleosol surface, rock hammers and a chisel were used to trench to a depth of 100 cm at a horizontal angle into the outcrop. Large (~ 0.25 kg), lithified, brick-like samples were collected at depths of 0 cm (paleosol surface), 25 cm, 50 cm, 75 cm and 100 cm, wrapped in ashed aluminum foil, and immediately placed into a cooler for ~ 8 h before transport into a − 80 °C freezer.

### Laboratory analyses

Three independent laboratory analyses were performed to examine soil organic carbon (SOC) pools in the paleosol samples. First, we measured SOC abundances in bulk samples to determine if the samples contained sufficient quantities of organic carbon for radiocarbon dating. We then performed radiocarbon dating of bulk samples to identify any additions of recent/modern (radiocarbon) organic carbon to the SOC pool. Third, we evaluated the thermal stability of SOC using thermal and evolved gas analysis as a proxy for recalcitrance and dynamics of organic compounds in bulk samples^6,26^. By monitoring evolutions of H_2_O from phyllosilicate dehydroxylation and co-occurring releases of CO_2_ and organic fragments from organic carbon decomposition, we determined if organic molecules were associated with clay minerals and/or sulfate minerals in several of the samples.

### Total organic carbon determination and radiocarbon (^14^C) dating

All samples for total organic carbon determination and radiocarbon dating were acid-washed to remove inorganic carbon. To test the hypothesis that paleosols contain organic C that is radiocarbon-dead, we chose acid washing^15^ rather than acid fumigation^32^ of samples for removal of inorganic C. Acid-washing samples presumably leads to the removal of some or all of the soluble organic C from bulk samples^33^, which may have removed both modern and ancient soluble C materials. If some amount of radiocarbon is detected after acid washing, this would imply that any radiocarbon may have been composed of insoluble C compounds that were, for example, associated with clay minerals or amorphous/nanocrystalline phases^34^.

For acid washing, ground paleosol samples (~ 5 g) were treated with approximately 20 mL of 0.1 M HCl at room temperature for 1 h, washed three times with ~ 30 mL of deionized water and then dried at 60 °C for 24 h. Samples were then manually encapsulated in 5 × 8 mm tin capsules (sample size approximately 0.25–0.70 mg). Total organic carbon was determined by elemental analysis on a Costech ECS 4010 instrument at the University of Oregon with expected SD < 0.3%^35^. The TOC in each sample was calculated to optimize aliquot amounts for radiocarbon dating. All samples were analyzed in duplicate.

A conventional radiocarbon age^36^ of organic carbon was obtained from ten samples across three paleosol profiles. Radiocarbon dating of acid-washed paleosol samples was performed at the W.M. Keck Carbon Cycle Accelerator Mass Spectrometer at the University of California, Irvine. An additional TOC determination was performed at UC Irvine and is reported in Table 2. The accuracy and precision (1 σ) of this analysis on modern carbon (Δ ^14^C > 0‰) was better than 1.5‰. Laboratory radiocarbon blanks yielded a Δ ^14^C value of − 996.2‰. Calibration of conventional radiocarbon dates was performed using the OxCal calibration^37^ with calibrated dates and errors reported in Table S3.

### Quantifying additions of modern carbon to bulk paleosol samples

We used an isotopic mixing model to estimate the relative proportion of recent/modern carbon in bulk paleosol samples. Based on the distinct isotopic composition of modern organic carbon and radiocarbon-free carbon, a two-endmember mixing model^21,22^ was used to quantify the relative proportions of modern and ancient carbon as distinct sources of the paleosol organic carbon pool^14^. We used the following equation to partition modern (post-bomb) organic carbon (Δ ^14^C = 0‰ to 600‰) from radiocarbon-dead organic carbon (Δ ^14^C ~  − 1000‰):

1 $${\text{C}}_{{{\text{modern}}}} = {\text{ C}}_{{\text{t}}} \left( {\Delta^{{{14}}} {\text{C}}_{{{\text{bulk}}}} - \, \Delta^{{{14}}} {\text{C}}_{{{\text{Oligocene}}}} } \right)/\left( { \, \Delta^{{{14}}} {\text{C}}_{{{\text{Modern}}}} - \, \Delta^{{{14}}} {\text{C}}_{{{\text{Oligocene}}}} } \right)$$

where C_t_ is the total amount of organic carbon (TOC) measured in bulk samples, Δ ^14^C_bulk_ is the measured Δ ^14^C value of bulk samples, Δ ^14^C_Modern_ is a typical value for a modern post-bomb organic carbon endmember, where three scenarios for the Δ ^**14**^**C** of modern carbon were considered (Δ ^**14**^**C** = 0‰, 300‰ and 600‰), Δ ^**14**^**C**_Oligocene_ is a typical Δ ^**14**^**C** value for a radiocarbon-free organic carbon endmember (Δ ^**14**^**C** = approximately − 1000‰), and C_modern_ is the modelled fraction of modern organic carbon in bulk samples. We considered model outputs with two values for the modern endmember (0‰, 300‰ and 600‰) to capture potential variability of modern (post bomb) C additions from labile and recalcitrant C pools^38^. Errors were propagated to estimate uncertainty associated with modelled values. The sources of uncertainty considered in the model were (a) the uncertainty of the measured TOC values; (b) uncertainty of the measured Δ ^**14**^**C** values; and (c) uncertainty of model predictions when “modern” C was assumed to be 0‰, 300‰ or 600‰.

To test the hypothesis that mixing of modern (Δ ^14^C ~ 0‰) and Oligocene (28–33 Ma) carbon caused the measured radiocarbon ages, we took a Keeling plot approach^39^ to estimate the ^14^C signal of the old-carbon endmember in paleosols. Applications of Keeling plots to terrestrial carbon dynamics can help quantify the processes controlling ecosystem-scale isotopic discrimination^39^ and here may indicate the isotopic composition of the “old” carbon endmember. We considered ^14^C isotope ratios (represented as FM, fraction modern) and the inverse of total organic carbon (TOC) and performed least-squares regressions of the fraction modern (FM) versus 1/TOC. In this way, the intercept represents the FM of the old-carbon endmember^39^. Intercept values that are approximately zero indicate that the old-carbon endmember is near radiocarbon-dead (e.g., older than ~ 45 Ka).

### Thermal and EVOLVED gas analysis

We used thermal and evolved gas analysis (EGA) to examine two out of the ten samples that were radiocarbon dated. Thermal and evolved gas analysis is an analytical technique for characterizing the organic and mineral content of natural soil and sediment samples^24^ and was used here for examining the thermodynamic stability of C pools in bulk samples. During EGA, ramped combustion from 35 to 1000 °C generates a time and temperature curve for each volatile gas (e.g., CO_2_, H_2_O, SO_2_) released during the thermal decomposition of the sample. This technique constrains the amount and thermodynamic stability of organic carbon in bulk samples^40^, as well as revealing if organic carbon was associated with phyllosilicates or other minerals^27,41^.

The peak release temperature of CO_2_ from organic carbon decomposition can reveal differences in the thermodynamic stability of organic carbon compounds in SOC pool^23^. Because there are large differences in the thermal stability between labile and recalcitrant organic carbon compounds^24,42^ it is possible to constrain the thermodynamic stability (e.g., resistance to oxidation) of organic carbon. This can be used to help evaluate whether paleosol samples contain significant amounts of labile modern organic carbon that could have originated in the modern weathering zone. Samples for EGA were not acid-washed before analysis because ramped EGA takes advantage of the large differences in thermal stability of organic and inorganic carbon to simultaneously examine both carbon pools in bulk soil samples.

We also used EGA to determine if organic compounds in paleosols were primarily associated with pedogenic minerals. Since paleosols are known to preserve autochthonous organic carbon in association with clay mineral surfaces for hundreds of millions of years^3,43^, it is important to determine if the Oregon paleosols contain organic carbon, whether ancient or modern, that is associated with clay minerals or other volatile-bearing phases. Many of the paleosols examined in the present study contained between ~ 70 and 95 wt% Al and Fe smectites, primarily as mixtures of montmorillonite and nontronite^30^. Therefore, thermal analysis of bulk samples and their inherently high clay content could help to constrain if organic molecules are present in association with clay minerals.

Sulfur-bearing phases may also have contributed to organic preservation in paleosols and are potentially detectable with EGA. Past work determined that several of the samples analyzed here contained trace amounts of sulfate minerals (gypsum and jarosite) that were likely inherited from the modern weathering zone and not original to the paleosol^30^. During EGA, simultaneous releases of SO_2_ and CO_2_ would indicate that organics, whether ancient or modern, could have also been associated with sulfate minerals (e.g., sorbed or occluded), or preserved as organo-sulfur compounds through abiotic reactions such as sulfurization of organic matter^44–46^.

A Setaram Labsys Evo differential scanning calorimeter/thermal gravimeter connected to a Pfeiffer Omnistar quadrupole mass spectrometer was used for thermal and evolved gas analysis. All analyses were performed in the Mars, Moon and Meteorite evolved gas analysis laboratory at NASA Johnson Space Center. Approximately 50 mg ± 3 mg of ground paleosol sample was placed in an Al_2_O_3_ sample crucible (previously ashed at 550 °C before introduction of the sample). The sample crucible and an identical empty reference crucible were placed in the furnace. The instrument was then purged twice with helium to remove any contamination in the system, then set to a pressure of 3 kPa He prior to sample analyses. Helium was chosen as a carrier gas because it is inert. The crucibles containing samples were heated from approximately 35 °C to 1000 °C at a heating rate (ramp rate) of 35 °C/min and with a helium flow rate of 10 cm^3^/s. A series of three blanks were analyzed before and after each group (n = 10) of samples. Volatiles ranging from mass/charge (*m/z*) 1–100 were measured. All analyses were performed in duplicate. All sample runs were background corrected. Raw EGA data is included as supplementary material.

## Results and discussion

Total organic C, measured in three individual profiles spanning depth transects from the outcrop surface to a 1-m depth, ranged from 0.01 to 0.3 wt% and had no clear C-concentration or age-depth profile (Table 1). Despite acid pretreatment to remove carbonates and potentially soluble modern C, all samples contained radiocarbon. Ten samples from three different paleosol profiles showed Δ ^**14**^**C** values ranging from − 768.3‰ ± 1.3‰ to − 971.9‰ ± 0.9‰ and conventional radiocarbon ages from 11,750 ± 50 years BP to 30,110 ± 320 years BP (Table 1). The fraction of modern carbon (FM) ranged from 0.0236 ± 0.008 to 0.2333 ± 0.013 (Table 1) and was highest in the Luca profile and lowest in the Turtle Cove profile (Fig. 2). One of the pedotypes (Luca) is a highly oxidized paleosol with very low TOC; thus, only one sample from this profile was able to be radiocarbon-dated, and this sample required combustion of over 1 g of material (Table S2). Calibrated radiocarbon dates ranged from 11,550 cal years BP to 33,322 cal BP (Table S3).

**Table 1:** Total organic carbon and uncalibrated radiocarbon dates of ten paleosol samples from eastern Oregon.

Sample	Depth (cm)	Age (Ma)	TOC (wt%)	Δ ^14^C	Error ±	^14^C age (Yrs BP)	Error ± (Yrs)
Lakim	0	~ 33	0.302	− 943.5	0.9	23,020	130
Lakim	25	~ 33	0.116	− 865.4	1.0	16,040	70
Lakim	50	~ 33	0.128	− 921.1	0.9	20,340	90
Lakim	75	~ 33	0.251	− 945.8	0.9	23,340	140
Lakim	100	~ 33	0.132	− 820.7	1.2	13,740	60
Luca	0	~ 33	0.060				
Luca	22	~ 33	0.014	− 770.3	1.3	11,750	50
Luca	50	~ 33	0.005				
Luca	75	~ 33	ND^Ʊ^				
Luca	100	~ 33	0.001				
TC	0	~ 28	0.170	− 972.2	0.9	28,700	260
TC	25	~ 28	0.196	− 971.8	0.8	28,590	230
TC	50	~ 28	0.261	− 976.6	0.9	30,110	320
TC	100	~ 28	0.115	− 938.5	0.9	22,330	120

Depth (cm) represents the horizontal depth into the outcrop from where samples were gathered. TOC, total organic carbon, measured at University of Oregon; Ma, Millions of years ago. The estimated age of the outcrop (“Age”) was previously determined by ^40^Ar-^39^Ar dating (Bestland, 1997) and is distinct from ^14^C age (years BP). Calibrated radiocarbon ages are reported in Table S3.

ND^Ʊ^, below limit of detection.

Two hypotheses to explain the conventional radiocarbon dates are A) additions of modern organic carbon to bulk samples (e.g., a Δ ^**14**^**C** ~ 0‰ modern carbon pool mixing with an ancient, radiocarbon-free pool); or B) a Pleistocene (~ 11–30 Ka) productivity event which introduced carbon into the paleosols (e.g., the carbon is indeed tens of thousands of years old). An additional possibility is that C compounds are older than 50–60 Ka but younger than Oligocene, which would suggest the addition of C that is allochthonous to paleosols but outside of the range of radiocarbon dating techniques (approximately 50,000–60,000 years BP).

Lithified hand samples of the Turtle Cove soils commonly contain carbonaceous root traces, suggesting the preservation of Oligocene fossil plant material^9^. Recent work has also shown that large abundances of amorphous colloids (~ 40 wt%) persist in Turtle Cove paleosols^34^. Nanocrystalline and/or amorphous phases are not expected to survive burial diagenesis^9^, so their presence is unanticipated in ~ 28 Ma Andisol and Aridisol paleosols buried by 1–2 km of overburden^34^. As in modern soils, autochthonous organic C in paleosols may be associated with amorphous or nanocrystalline materials that were original to the soil, such as allophane and/or imogolite^9^, if these phases survived burial diagenesis. In modern soils, reactive amorphous phases have a high affinity for sorption of organic molecules^47,48^, which can increase the proportion of recalcitrant C compounds in bulk samples^49^. Though possible, it is unclear if a similar process occurred in ancient samples examined here, but it is apparent they contain measurable amounts of radiocarbon.

One likely source of radiocarbon may be precipitation-driven leaching of dissolved organic carbon from modern biota living in the current weathering zone above paleosol outcrops (Δ ^**14**^**C** ~ 0‰). As such, it is possible that small amounts of modern organic carbon from the weathered zone above paleosol outcrops have mixed with larger amounts of ^14^C-free organic carbon that is endogenous (e.g., autochthonous) to paleosols. In this way, a radiocarbon date of ~ 11,000–30,000 years BP could represent a mixing of modern organic carbon and 33 Ma organic carbon.

This hypothesis is supported by the rapid erosion rate for the site, which was previously determined to be approximately 4.94 ± 0.05 mm/year^50^. Using this erosion rate, the ~ 20 cm-thick soils that formed on top of the paleosol outcrops are only about 40 years old and could have leached modern organics into the underlying paleosols during this time. This rapid erosion rate indicates that the mantling soils may be only tens of years old and thus may have a large proportion of modern C compounds. It also is therefore unlikely that Pleistocene (~ 11–30 Ka) relict soils atop profiles are a source of radiocarbon because at such rapid erosion rates these soils would have presumably been eroded away long ago.

Application of a two-endmember isotopic mixing model to the measured Δ ^14^C values (Eq. 1) for estimation of modern organic carbon abundances in bulk paleosol samples is shown in Table 2. The modelled abundances of modern carbon (assuming modern carbon was 0‰) ranged from 0.46% ± 0.19% to 2.36% ± 0.19% of the total organic carbon in each sample. Lower estimates of modern carbon abundance (0.20 ± 0.19 to 1.46 ± 0.19) were observed when modern carbon was assumed to be 300‰ and 600‰ (Table S4). We also evaluated the old carbon endmember as ~ 11 Ka (− 776‰) and ~ 21 ka (− 930‰)^51^ instead of − 1000‰ (Table S4) with observed modelled C values ranging from ~ 1.5 to 10.5% for ~ 11 Ka carbon and ~ 4.6 to 33.6% for ~ 21 Ka carbon. It is important to note that all modelled values may be a fundamental underestimation of the total amount of radiocarbon accumulation in these samples because only the acid-insoluble fraction of C compounds were examined here (see Methods). Altogether these results support the hypothesis that the measured Δ ^14^C values represent the mixing of small amounts of modern organic C with larger amounts of radiocarbon-free C.

**Table 2:** Application of a two-endmember mixing model to the measured Δ ^14^C values in ten paleosol samples.

Paleosol	Depth (cm)	TOC (wt%)	1/TOC	FM	Modern C (%)	Error ± (%)
Lakim	0	0.26	3.80	0.057	1.70	0.19
Lakim	25	0.09	11.21	0.136	1.56	0.19
Lakim	50	0.14	7.07	0.080	1.00	0.19
Lakim	75	0.24	4.23	0.055	1.36	0.19
Lakim	100	0.03	30.86	0.181	2.36	0.19
Luca	22	0.01	71.43	0.232	0.32	1.84
TC	0	0.17	5.87	0.028	0.47	0.19
TC	25	0.15	6.80	0.028	0.55	0.19
TC	50	0.23	4.35	0.024	0.61	0.19
TC	100	0.08	11.82	0.062	0.71	0.33

TOC, Total organic carbon, measured at UC Irvine; FM, Fraction modern; Modern C, modelled abundance of modern carbon in bulk paleosol samples, representing the estimated percent of modern carbon within bulk samples.

### Constraining the ^14^C signal of old carbon in bulk paleosol samples with Keeling plots

**Figure 3:**
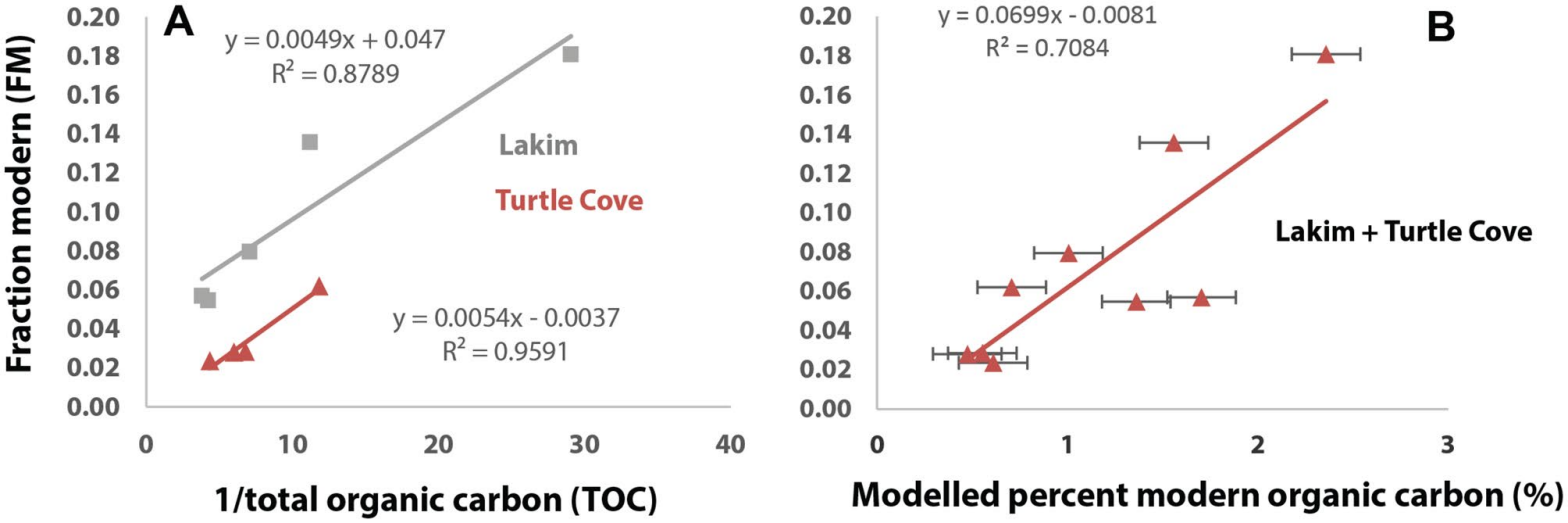
Keeling plots and a two-endmember isotopic mixing model to measured Δ ^14^C values of bulk paleosol samples. (**A**) A Keeling plot approach to estimate the ^14^C signal of the old-carbon endmember of two paleosol profiles where the ^14^C signal of the “old” carbon endmember is the y-intercept; (**B**) the relationship between fraction modern (FM) values from bulk samples and the modelled abundance of modern organic carbon. We assumed a Δ ^14^C value of 0‰ for modern carbon; model outputs for Δ ^14^C of 300‰ and 600‰ for modern C were also considered and are listed in Table S4.

Figure 3A shows the positive significant relationship between FM and 1/TOC in two of the paleosol profiles where the intercept is the Δ ^14^C signal of the old-carbon endmember. The modelled FM of the old-carbon endmember in the Lakim profile was 0.047 ± 0.0012, consistent with a near-radiocarbon dead endmember (intercept of 0), though with a greater proportion of potentially modern radiocarbon as inferred from the mixing model (Table 2). By contrast, the Turtle Cove profile had an intercept of 0.004 ± 0.0012, which was consistent with a near radiocarbon-dead endmember, suggesting this sample contained an C endmember that was free of radiocarbon.

The modelled FM values from Keeling plots agree with the modelled abundances of modern carbon in each of the paleosol profiles (Table 2). The profile with the greatest modelled amount of modern carbon (Lakim) also had the highest FM, while the profile with the least amount of modern carbon (Turtle Cove) had a much older ^14^C signature for the old-carbon endmember, and thus a lower FM (Fig. 3B). Interestingly, the Turtle Cove profile also had relatively high TOC (~ 0.2 wt%), which indicates this paleosol could contain relatively large amounts of ancient C compounds, some of which may be Oligocene in age.

**Figure 4:**
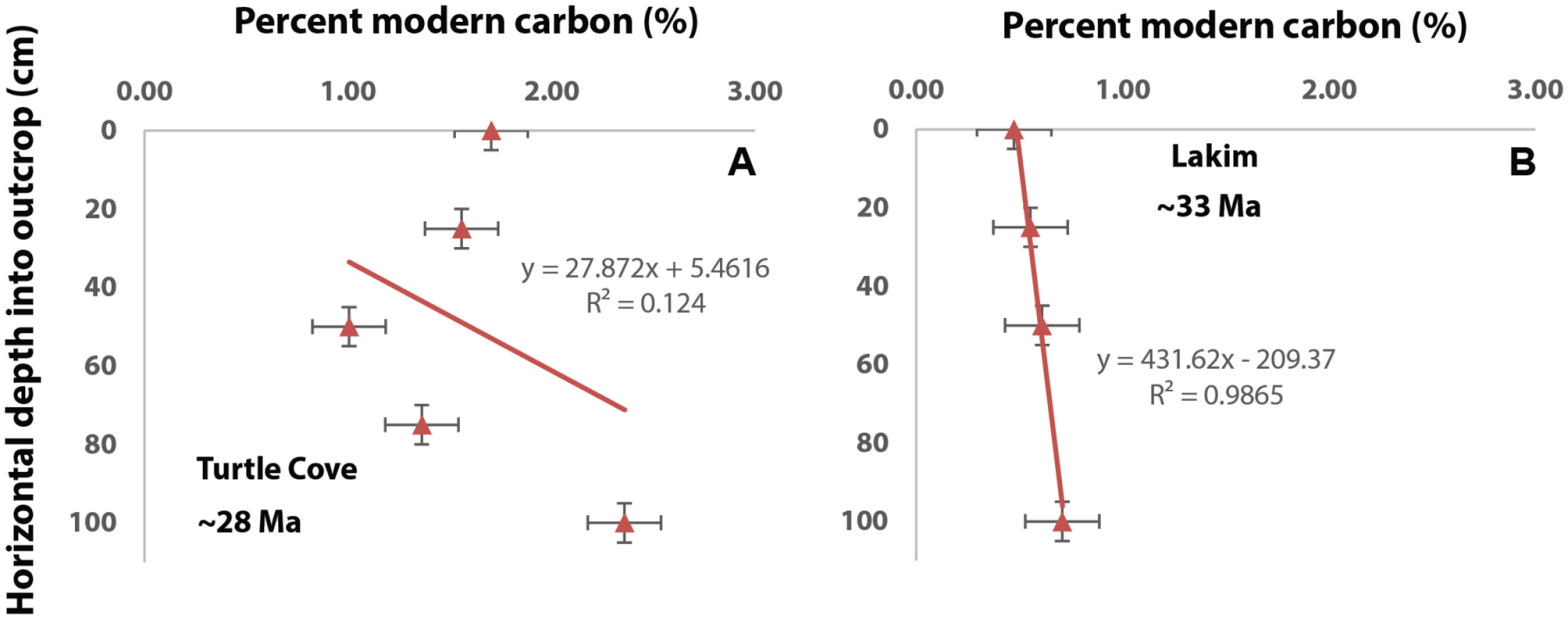
The relationship between sampling depth and the modelled percent of modern carbon in two Oligocene (28–33 Ma) paleosol profiles. (**A**), Turtle Cove paleosol; (**B**) Lakim paleosol (note only four samples were considered). Uncertainties for sampling depth (± 5 cm) and propagated error of model predictions for percent modern carbon (± 0.19%) are shown for each profile.

Figure 4 demonstrates radiocarbon accumulation a function of horizontal depth into the exposed outcrop. One striking observation in the Turtle Cove soil was the significant (*P* < 0.001) relationship between horizontal depth into the outcrop and the modelled percent of modern carbon (n = 4; R^2^ = 0.99). However, it should be noted that there were only four data points considered for the Turtle Cove soil, and thus these results should be interpreted with caution. The Lakim soil (n = 5) showed an erratic depth function (Fig. 3B), possibly because the 50 cm and 75 cm samples contained significantly (*P* < 0.05) less modern organic carbon than most other samples in the transect. Interestingly, the deepest samples in the two profiles (100 cm) contained the highest modelled abundances of modern organic carbon, which is contrary to expected accumulation in near-surface samples.

Although it is challenging to determine what specific biogeochemical processes may have led to the depth functions (Fig. 4), differences in porosity or topography of the sites could explain the relationship between depth and modern carbon abundance. The Luca and Lakim soils were rich in clay minerals (> 80 wt% smectite) with low porosity whereas the Turtle Cove soil contained less clay (< 50% wt%) and possibly a large amount of amorphous/nanocrystalline material^34^ which could have led to increased porosity. Higher porosity may be associated with increased leaching of recent/modern C compounds^9^. Additionally, all three paleosol profiles were located at a toeslope setting with differences in the degree of slope. The Lakim soil was trenched at a ~ 65° angle relative to the surface due to the gentle sloping nature of the outcrop (slope ~ 45°), whereas the Turtle Cove sample was trenched nearly perpendicular relative to the surface because the outcrop was a near-vertical wall (slope ~ 90°) (Fig. 2). This could have ultimately led to the observed radiocarbon trends in each of the soils. Alternatively, the precipitation-driven leaching of dissolved organic carbon could have infiltrated deep (> 1 m) into the matrix of the exhumed paleosols in an inherently heterogenous manner, such as along randomly oriented fractures in the lithified matrix.

Topography could have further influenced the leaching of dissolved modern organic carbon because of differences in permeability of overlying materials. The Turtle Cove outcrop was buried by several ash layers and then a relatively impermeable ignimbrite (Fig. 2) whereas the Lakim and Luca soils were buried by a biotite-bearing tuff, which presumably has higher permeability compared to the ignimbrite. Additional possibilities are eolian deposition and leaching of organic C or groundwater-driven accumulation of dissolved organic C, though there was no morphological evidence of modern groundwater alteration in any of the profiles examined.

Alternatively, it is worth considering that Pleistocene (11–30 Ka) carbon could have accumulated in the soils, possibly from leaching of dissolved organic carbon resulting from a Pleistocene productivity event. Previous work has shown that the field site was ice-free and adjacent to pluvial lakes during the late Pleistocene^18^. Eastern Oregon was characterized by highly productive pluvial lakes and grassland soils associated with Pleistocene megafauna (e.g., “Mammoth Steppe”)^52^. Therefore, it is possible that Pleistocene organic carbon leached into paleosols at that time, but we consider this hypothesis unlikely because of the rapid erosion rate for the site, such that remnants of Pleistocene (11–30 Ka) soil and carbon may have long ago been removed by erosion. Assuming a constant erosion rate of ~ 4.9 mm/yr.^50^, approximately 54 m of overburden could have been eroded from the site in the past 11,000 years.

### Thermal and evolved gas analysis (EGA)

**Figure 5:**
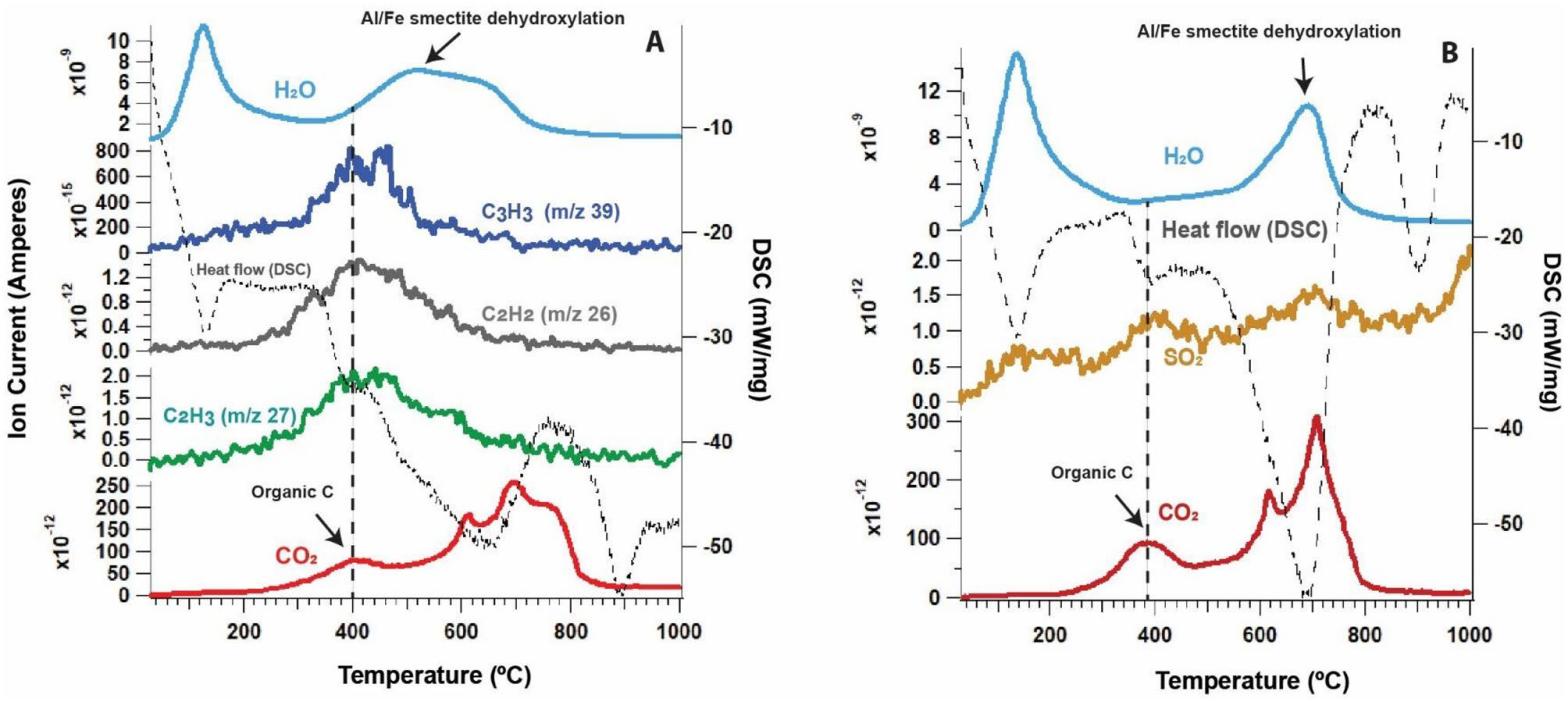
Thermal and evolved gas analysis of two paleosol samples from the early Oligocene (33 Ma) Big Basin Member of the John Day Formation (adapted from ^31^). Dashed vertical line at 400 °C in both panels is from the thermal decomposition of organic carbon. H_2_O—*m/z* 18, SO_2_—*m/z* 64, CO_2_—*m/z* 44. (**A**), Evolutions of CO_2_, organic fragments (C_2_H_2_, C_2_H_3_, C_3_H_3_), and H_2_O from the surface horizon of the Luca paleosol. Dashed trace is heat flow from differential scanning calorimetry (DSC) where exothermic reactions have a positive slope; (**B**) Evolutions of H_2_O (blue trace), SO_2_ (yellow trace) and CO_2_ (red trace), from a thin Entisol (“Kskus” pedotype) stratigraphically below the Luca profile^30^. Overlap of SO_2_ and CO_2_ peaks at 400 °C in B) suggests sulfur-bearing phases (possible sulfides) may also associated with organic carbon.

Figure 5 shows the thermal and evolved gas analysis (EGA) of two paleosol samples. The DSC and volatile curves were representative of a complex pedogenic mineral mixture, with many different exothermic and endothermic reactions simultaneously occurring over a range of temperatures. Evolutions of CO_2_ from organic carbon decomposition had a consistent peak release temperature at ~ 400 °C which tracked with a small exotherm, suggesting the presence of recalcitrant organic carbon with resistance to low-temperature (150–300 °C) oxidation (Fig. 5). Evolutions of organic fragments (C_2_H_2_, C_2_H_3_ and C_3_H_3_) co-occurred with the release of CO_2_, which provided further evidence that the CO_2_ peak at 400 °C was from organic carbon because these molecules are byproducts of thermal decomposition of organic matter^53^. On the other hand, high-temperature (> 550 °C) evolutions of CO_2_ in paleosol samples (Fig. 5) that tracked with an endotherm were instead consistent with the thermal decomposition of inorganic carbon (e.g., small amounts of Ca carbonate). Similar organic C-CO_2_ peak release temperatures of 400–500 °C were previously noted in the Pleistocene “Brady” paleosol from Nebraska and were attributed to the preservation of recalcitrant organic carbon such as black carbon (char) and plant lipids^6^.

Samples for EGA were not acid washed (see Methods) because acid pretreatment steps to remove carbonates can alter original quantities of TOC in samples^40^. Thus, it is possible that small amounts of carbonates were present in our samples. However, soil organic matter that is associated with clay minerals can also have CO_2_ release temperatures that overlaps with the CO_2_ release from inorganic carbon in the ~ 550–700 °C range^40,41^, primarily because interactions with mineral surfaces can increase the thermodynamic stability of organic carbon, which can lead to an increase in the temperature of thermal decomposition during pyrolysis^26^. In our samples, mineral-associated organic carbon could have been responsible for the high temperature (~ 700 °C) exothermic CO_2_ peaks observed in both samples (Fig. 5, red trace). Alternatively, this CO_2_ peak could have resulted from the thermal decomposition of refractory organic carbon such as kerogen^27^, or from the decomposition of inorganic C. Since these ancient soils were buried by an estimated 1–2 km of overburden^9^, refractory organic compounds could have formed as a result of burial and diagenesis^10^.

From the EGA data alone, there was no clear evidence that organic carbon was predominantly associated with clay minerals (Fig. 5, blue and red traces). The peak release temperature of CO_2_ and organic fragments from organic carbon decomposition at 400 °C (vertical dashed line at 400 °C, Fig. 5) was offset by approximately 100 °C from the peak H_2_O release from phyllosilicate dehydroxylation (~ 500–650 °C). If organic molecules were strongly associated with clay mineral surfaces and/or interlayer spaces, there would be little or no apparent offset between CO_2_ and H_2_O during heating (e.g., the two peaks would present as co-evolving).

In a paleosol sample taken from directly below the Luca soil profile (Kskus pedotype from^31^) we also observed small amounts of evolved SO_2_ that co-occurred with organic carbon decomposition at ~ 400 °C (yellow trace, Fig. 5b), suggesting the presence of organo-sulfur compounds^54^ or the decomposition of a sulfide mineral^55^. Sulfur-bearing phases such as gypsum and jarosite were previously observed at the Luca field site in small quantities and most likely formed in the gypsic Aridisols of the modern weathering zone^30^. Abiotic sulfurization of organic matter can also enhance organic preservation in marine sediments^45^ and paleosols^5^. Thus, sulfur-bearing phases from the modern weathering zone could have also contributed to the preservation of carbon in paleosols from the field site.

Though the EGA results do not provide direct evidence that phyllosilicates and organics were strongly associated with one another, it is possible that the high clay mineral abundances provided other means of organic preservation, including physical occlusion, the formation of organic-mineral aggregates, or other weaker types of sorption to phyllosilicate surfaces, such as outer-sphere complexation^47,56^ which could have resulted in the organic carbon decomposing at temperatures ~ 100–200 °C lower than clay mineral dehydroxylation. Physical soil fractionation to concentrate clay minerals and associated organics may provide more useful results than EGA of bulk samples as performed here, though may prove challenging because of the lithification and diagenetic alteration that have acted upon ancient soil samples. In any case, an evaluation of mineral-associated organic carbon content using conventional methods (e.g.,^6^) would be useful to confirm the EGA results presented here. These results illustrate the complexity of the organic carbon pool within ancient, buried soils, and they also demonstrate the fundamental limitations of pyrolysis methods such as EGA for constraining the organic and inorganic C content of natural soil samples.

## Conclusion

Radiocarbon analysis of samples from 28 to 33 Ma paleosols in badland landscapes that are separated by time and space showed widespread accumulation of radiocarbon deep within lithified layers. Conventional radiocarbon ages of all samples ranged from ~ 11,000 to 30,000 years BP. We hypothesized these dates represent a mixing of modern C with larger amounts of radiocarbon-dead C. The highest amounts of recent/modern C were noted in the deepest samples (1 m depth) rather than samples from the surface of outcrops, potentially suggesting heterogenous and erratic accumulation of radiocarbon. These results indicate paleosols readily interact with the modern carbon cycle via the addition of small amounts of radiocarbon. This may also imply that paleosols may contribute to modern C cycling via the oxidation of autochthonous paleosol C by microbes in the modern weathering zone.

Application of a two-endmember isotopic mixing model based on measured Δ ^**14**^**C** values of bulk samples suggested that modern organic carbon comprised approximately 0.46% to 2.36 ± 0.19% of the paleosol organic carbon pool. A Keeling plot approach to determine the ^14^C signature of the old-carbon endmember in paleosols also suggested a mixing of modern carbon and potentially Oligocene (radiocarbon-free) carbon. The modelled fraction modern (FM) of the old-carbon endmember in two paleosol profiles ranged from 0.004 ± 0.0012 to 0.047 ± 0.0012, both of which are consistent with a near radiocarbon-dead old-carbon endmember. Alternatively, the old carbon endmember could be Pleistocene (11–30 Ka), but the rapid erosion rate characteristic of these badland landscapes (> 4 mm/year) suggests that the 20-cm-thick modern soils mantling paleosol outcrops are approximately 40 years old, thus rendering the preservation of Pleistocene soils and carbon unlikely. Therefore it is possible that paleosols contain ancient radiocarbon-dead C compounds that are potentially Oligocene, and that the measured radiocarbon dates in bulk paleosol samples represent a mixing of modern (Δ ^14^C ~ 0‰) and ancient carbon (− 1000‰).

Thermal and evolved gas analysis was used to constrain the thermodynamic stability of organic carbon and to determine if organic carbon was primarily associated with clay minerals. A CO_2_ peak release temperature at 400 °C that co-occurred with evolutions of organic fragments was consistent with the presence of recalcitrant organic carbon, but there was no conclusive evidence that organic C, whether ancient or modern, was strongly associated with phyllosilicates because the peak release temperature of CO_2_ (from organic C decomposition) and H_2_O release (from smectite dehydroxylation) were offset by ~ 100–200 °C.

Although the sources and types of organic compounds remain unexplored, this work reveals the widespread accumulation of radiocarbon in lithified, brick-like paleosols that are millions of years old and suggests the accumulation of radiocarbon may be a common process within exhumed landscapes that host paleosol sequences. This work challenges the assumption that ancient badland landscapes are inert and frozen in time by revealing the amount of recent and/or modern C accumulation in paleosols, showing that such landscapes can interact with and contribute to the global carbon cycle.

## Supplementary Information

Supplementary Information 1.

Supplementary Tables.

## Data Availability

All data generated or analyzed during this study are included in this published article [and its supplementary information files].
